# A comparison of the Mini-Mental State Examination (MMSE) with the Montreal Cognitive Assessment (MoCA) for mild cognitive impairment screening in Chinese middle-aged and older population: a cross-sectional study

**DOI:** 10.1186/s12888-021-03495-6

**Published:** 2021-10-04

**Authors:** Xiaofang Jia, Zhihong Wang, Feifei Huang, Chang Su, Wenwen Du, Hongru Jiang, Huijun Wang, Jiaqi Wang, Fangjun Wang, Weiwu Su, Huifang Xiao, Yanxin Wang, Bing Zhang

**Affiliations:** 1 National Institute for Nutrition and Health, Chinese Center for Disease Control and Prevention, Beijing, 100050 China; 2 School of Public Health, Hebei Medical University, Shijiazhuang, 050017 China; 3 Yongkang Center for Disease Control and Prevention, Yongkang, 321300 China; 4 Yuelu District Center for Disease Control and Prevention, Changsha, 410013 China; 5 Changde Center for Disease Control and Prevention, Changde, 415000 China; 6 Shaanxi Provincial Center for Disease Control and Prevention, Xian, 710054 China

**Keywords:** Mild cognitive impairment, MMSE, MoCA, Correlation, Agreement, Risk factors

## Abstract

**Background:**

The Mini-Mental State Examination (MMSE) and the Montreal Cognitive Assessment (MoCA) are the most commonly used scales to detect mild cognitive impairment (MCI) in population-based epidemiologic studies. However, their comparison on which is best suited to assess cognition is scarce in samples from multiple regions of China.

**Methods:**

We conducted a cross-sectional analysis of 4923 adults aged ≥55 years from the Community-based Cohort Study on Nervous System Diseases. Objective cognition was assessed by Chinese versions of MMSE and MoCA, and total score and subscores of cognitive domains were calculated for each. Education-specific cutoffs of total score were used to diagnose MCI. Demographic and health-related characteristics were collected by questionnaires. Correlation and agreement for MCI between MMSE and MoCA were analyzed; group differences in cognition were evaluated; and multiple logistic regression model was used to clarify risk factors for MCI.

**Results:**

The overall MCI prevalence was 28.6% for MMSE and 36.2% for MoCA. MMSE had good correlation with MoCA (Spearman correlation coefficient = 0.8374, *p* < 0.0001) and moderate agreement for detecting MCI with Kappa value of 0.5973 (*p* < 0.0001). Ceiling effect for MCI was less frequent using MoCA versus MMSE according to the distribution of total score. Percentage of relative standard deviation, the measure of inter-individual variance, for MoCA (26.9%) was greater than for MMSE (19.0%) overall (*p* < 0.0001). Increasing age (MMSE: OR = 2.073 for ≥75 years; MoCA: OR = 1.869 for≥75 years), female (OR = 1.280 for MMSE; OR = 1.163 for MoCA), living in county town (OR = 1.386 and 1.862 for MMSE and MoCA, respectively) or village (OR = 2.579 and 2.721 for MMSE and MoCA, respectively), smoking (OR = 1.373 and 1.288 for MMSE and MoCA, respectively), hypertension (MMSE: OR = 1.278; MoCA: OR = 1.208) and depression (MMSE: OR = 1.465; MoCA: OR = 1.350) were independently associated with greater likelihood of MCI compared to corresponding reference group in both scales (all *p* < 0.05).

**Conclusions:**

MoCA is a better measure of cognitive function due to lack of ceiling effect and with good detection of cognitive heterogeneity. MCI prevalence is higher using MoCA compared to MMSE. Both tools identify concordantly modifiable factors for MCI, which provide important evidence for establishing intervention measures.

## Background

Dementia is a leading cause of disability in people older than 65 years worldwide, including China, which induces huge challenges for policy makers, healthcare professionals, and family members [1]. Considering no effective treatment for dementia, as well as brain pathology which begins years before onset of objective cognitive symptoms and may be irreversible by the time of diagnosis, many investigators have shifted their focus toward delaying dementia in persons who are in preclinical phases of the disease. Mild cognitive impairment (MCI), referring to cognitive decline from a previous level of functioning both subjectively and by objective evidence, represents the preclinical, transitional stage between healthy cognitive aging and dementia, and affects 10–15% of the population over the age of 65 [2]. Although 20–30% of persons with MCI will revert to normal at subsequent follow-up [3], there is a 5–10% annual rate of progression to dementia in those with MCI, which is much higher than the 1–2% incidence per year among the general population [4]. Moreover, it has been suggested that approximately 50% will progress to dementia in 5 years [3]. MCI represents what researchers and clinicians regard as a “window” in which it may be possible to intervene and delay development to dementia [2]. It is thus imperative to screen for MCI and clarify potential influencing factors for MCI in old population at risk in large-scale study in efforts to improve cognitive functioning and delay progression to dementia.

In addressing cognitive screening tools, the Mini-Mental State Examination (MMSE) and the Montreal Cognitive Assessment (MoCA) are the most commonly used methods in cognitive impairment detection in both clinical and research fields [5–8]. It was widely identified that MoCA was superior to MMSE in the detection of MCI as the MMSE had lower sensitivity among multiple study settings [9–13]. Furthermore, the MoCA showed differences in cognitive profile even in those performing in the normal range on the MMSE and would appear to be a useful brief tool to assess cognition in those with MCI, particularly where the ceiling effect of the MMSE is problematic [8, 14, 15]. Similar studies were carried out in China, however, these studies were done in single region with small sample size, thereby suffered from a lack of representation and reliablity [16, 17] and studies in particular to compare the MMSE and MoCA in the detection of MCI among community-based samples are rare. Therefore, studies in multiple regions are further warranted to confirm the concordance between MMSE and MoCA in the identification of MCI, which may yield different and novel findings because of large sample size.

Additionally, to understand potential and modifiable risk factors to cognitive complaint is to some extent crucial for defense, treatment and intervention in the precarious state of MCI, thereby may delay progression to dementia. Researches to date have identified several factors, such as age, gender, educational and occupational attainment, marriage, income, psychological well-being, physical exercise, social engagement, diet and history of chronic diseases [18–22], but some of these findings were controversial, which might be attributable to varied countries of study origin, and the heterogeneity in research methods, including the age range included and the use of different cognitive assessment methods and diagnostic criteria. Especially education had strong influence on MMSE and MoCA performance [23, 24], and the unpredictable effects of those with more education performing poorer relative to those with less education was observed [24]. It is also necessary to distinguish whether there is disparity in potential factors for cognition when applying different cognitive screening tools to the same population.

Taken together, present study aims to determine the correlation and agreement between MMSE and MoCA in detecting MCI, and to test their differences in influencing factors for MCI among Chinese middle-aged and older population attending baseline survey of the Community-based Cohort Study on Nervous System Diseases in urban and rural areas of four provinces. Findings in this study may yield profound implications for the selection of cognitive measures and MCI management.

## Methods

### Study population

Data in the present study were derived from the baseline of the Community-based Cohort Study on Nervous System Diseases, an ongoing and longitudinal study established in 2018–2019 by National Institute for Nutrition and Health, Chinese Center for Disease Control and Prevention, which focused on potential factors associated with risks of three nervous diseases, including epilepsy for subjects aged > 1 year, and Alzheimer’s disease (AD) and Parkinson’s disease in ≥55 year-old population [25]. Participants without such diagnosed diseases at enrollment were recruited using a multistage stratified random sampling approach in Hebei, Zhejiang, Shaanxi and Hunan province, respectively. Two cities and two counties were randomly selected in each province. Urban and suburban neighborhoods within the cities, and townships and villages within the counties were selected randomly. In each community, all members meeting the inclusion and exclusion criteria of any of three nervous diseases in a randomly selected household were interviewed [25]. Protocol of this project was reviewed and approved by Medical Ethics Committee of National Institute for Nutrition and Health, Chinese Center for Disease Control and Prevention (No. 2017020, 6 November 2017). And written informed consent was obtained for each participant.

Present study targeted at subjects recruited in the cohort of AD. The eligible samples for inclusion were (1) 55 years old and older, (2) resident population living in the sampled community, (3) absent of clinically diagnosed AD, and (4) free of comorbid conditions that could affect assessment, such as congenital or acquired mental retardation, diagnosed MCI, and visual/hearing abnormalities even after correction [25]. Subjects with completed data of sociodemographic characteristics, disease history, cognitive examination, psychological evaluation, and survey of basic abilities of daily living were selected to participate in the present study. According to the definition of MCI, we excluded subjects because of their inability to perform basic activities of daily living involving eating, dressing, bathing, toileting, grooming, transferring bed or chair, walking across a room, and urinary or fecal continence (*n* = 71). For participants locating in the part of <P_1_ or > P_99_ (P: percentile) of sleep duration distribution in each age group, we used the corresponding P_1_ and P_99_ to replace those of <P_1_ or > P_99_, respectively. Finally, a total of 4923 participants were involved in the analysis.

### Cognitive assessment

All participants underwent cognitive assessment using Chinese version of the MMSE and the MoCA in present study. Both instruments were valid and reliable among Chinese by taking cultural and linguistical differences into account [26, 27]. MMSE and MoCA were conducted strictly face to face following the guidelines and protocols by trained investigators and were completed during 5–10 min and 10–15 min, respectively.

The MMSE is a 30-point questionnaire used extensively in clinical and research settings to measure cognitive impairment, including simple tasks in a number of areas: the test of time and place, the repeating lists of words, arithmetic such as serial subtractions of seven, language use and comprehension, and basic motor skills [7]. The MoCA is another 30-point test covering eight cognitive domains, and details on the specific MoCA items had been introduced by Nasreddine et al. [8]. The cultural and linguistic modifications of MoCA Beijing version we used from the original English version were also concretely described [27].

Cognitive function of different domains were evaluated according to items of each test [26]. Details on the components and corresponding maximum scores for each domain were shown in Table 1. The sum of included item points was the subscore of cognitive domain. Dysfunction of cognitive domain was defined as any incorrect test of included items, and cutoffs were listed in Table 1 [28].

**Table 1:** Cognitive domains assessed by the MMSE and MoCA

Domains	MMSE		MoCA	
	Items/maximum scores	Cutoffs of dysfunction	Items/maximum scores	Cutoffs of dysfunction
Orientation	Orientation to time and place/10	≤9/10	Orientation to time and place/6	≤5/6
Executive function	3-step command test/3	≤3/4	Trail-making test/1	≤5/6
	Reading command test/1		Digit span test/2	
			Verbal fluency/1	
			Abstraction test/2	
Calculation	Serial 7 substractions/5	≤4/5	Serial 7 substractions/3	≤2/3
Naming	Naming (pencil, cellphone)/2	≤1/2	Naming (lion, giraffe, camel)/3	≤2/3
Repetition	1 short sentence/1	0/1	2 longer sentences/2	≤1/2
Visuoconstructional skills	Copy intersecting pentagons/1	0/1	Copy cube/1	≤3/4
			Draw clock face/3	
Registration	Repeat 3 words/3	≤2/3		
Recall	Recall 3 words/3	≤2/3	Recall 5 words/5	≤4/5
Writing	Write a sentence/1	0/1		
Attention			Vigilance test for number ‘1’/1	0/1

The sum of all item points produced total scores of MMSE and MoCA, respectively, ranging from 0 to 30. A higher score indicates better cognitive function. When the education years of the participants were no more than 12 years, 1 point was added on their MoCA total score (if < 30) [8]. MCI was identified using education-specific cutoff points of total scores of MMSE and MoCA, respectively. MMSE ≤19 for illiterate individuals, ≤22 for participants with elementary school education, and ≤ 26 for those with middle school education and above [29]. According to Chinese MoCA norms [27], ≤13 for illiterate individuals, ≤19 for individuals with 1–6 years of education, and ≤ 24 for those with 7 or more years of education.

### Sociodemographic and health-related characteristics

Questionnaires were used to collect information on age, gender, educational level, current employment status, household income, residence area, current smoking, alcohol intake during last year, sleep duration covering daytime napping and full-night sleep, and disease histories of hypertension, diabetes, stroke and myocardial infarction by trained investigators. Additionally, a self-report assessment to identify depression in the elderly was performed using the Geriatric Depression Scale (GDS) 30-point version [30], and depression was defined if GDS score > 11 [31]. All these parameters were further grouped for data analysis (age: 55–64, 65–74 and ≥ 75 years; gender: male and female; educational level: below elementary school, elementary school, middle school, high school and above; monthly household income per capital: < 1000, 1000–3999, and ≥ 4000 Chinese yuan; statuses of current employment, current smoking and alcohol intake last year: yes and no; residence area: urban, suburban, county town, and village according to the administrative divisions; sleep condition: yes and no depend on if meeting age-specific sleep duration recommendations [32]; disease history of hypertension, diabetes, stroke or myocardial infarction: yes and no; and depression: yes and no).

### Statistical analysis

Continuous variables were presented as mean ± standard deviation (SD) and median, P_25_ and P_75_ were also calculated in order to evaluate presence of ceiling/floor effect in MMSE and MoCA tests, while categorical variables were expressed as n (%). Because of the non-normal distribution, non-parametric Wilcoxon rank-sum test or Kruskal-Wallis analysis was performed to test differences in distribution of MMSE or MoCA total score by sociodemographic and health-related factors. If the difference was significant among three subgroups and above, multiple comparison was conducted by Student-Newman-Keuls. The percentage for the relative standard deviation (RSD%) [(SD/mean) × 100] was calculated to examine inter-individual variance of the MMSE and MoCA total scores in the whole population, assuming that greater RSD% indicates better detection of cognitive heterogeneity of the sample [33]. The MMSE and MoCA RSD% index obtained were further compared by means of Wilcoxon signed-rank test. Prevalence of MCI by various factors was compared by Chi square test and Cochran-Armitage trend test if appropriate. And trends in proportions of subjects with MMSE-identified cognitive domain dysfunction across subscore strata of corresponding MoCA cognitive domain were also analyzed by Cochran-Armitage trend test. Scatter plot and Spearman correlation coefficient were applied to explore the correlation between MMSE and MoCA total scores. The agreement between MMSE and MoCA to detect MCI was obtained by Kappa value. Multiple logistic regression was employed, with MCI (yes vs. no) as dependent, and age, gender, employment status, household income, residence area, smoking, sleep condition, hypertension history and depression as independent variables, to explore the potential association of sociodemographic and health-related factors with MCI risk assessed by MMSE and MoCA, respectively. Predictors were simultaneously included in the regression model based on the significance of differences in MCI prevalence by studied factors in present study, and the evident influence of gender on MCI in previous studies [19, 34]. No collinearity between predictors was detected in both final models (tolerance: 0.79–0.99 and VIF: 1.01–1.27 for MMSE; tolerance: 0.80–0.98 and VIF: 1.02–1.26 for MoCA). A value of *p* < 0.05 was considered significant. Statistical analysis was carried out using SAS 9.4 (SAS Inc., Cary, NC, USA).

## Results

### Characteristics of study population

A total of 4923 subjects aged 55 years and more were included in this study (Table 2), in which those aged 55–64, 65–74 and ≥ 75 years accounted for 41.5, 40.7 and 17.8%, respectively. More than half of participants were female (56.1%). Around 18.2% of subjects completed the education of high school and above. The majority of participants were unemployed (82.8%), which included retired subjects. And the proportions of subjects with moderate monthly household income per capital and meeting the recommended age-specific sleep duration were 61.2 and 68.0%, respectively. People who smoked currently and drank alcohol last year accounted for 15.6 and 17.1%, respectively. The rates of people with reported disease history of hypertension, diabetes, stroke and myocardial infarction were 31.8, 9.7, 2.0 and 1.9%, respectively. And 8.6% subjects had self-reported depression in this study.

**Table 2:** Cognitive assessment by MMSE and MoCA by sociodemographic and health-related factors in study population

	N (%)	Score of cognitive assessment						Positive screening for MCI			
		MMSE		*p*-value	MoCA		*p*-value	MMSE		MoCA	
		mean ± SD	P_50_ (P_25_, P_75_)		mean ± SD	P_50_ (P_25_, P_75_)		n (%)	*p*-value	n (%)	*p*-value
Age group (years)				< 0.0001			< 0.0001		< 0.0001		< 0.0001
55–64	2042 (41.5)	26.5 ± 4.1	28 (25, 30)		23.8 ± 5.5	25 (20, 29)		496 (24.3)		650 (31.8)	
65–74	2004 (40.7)	25.5 ± 4.7	27 (23, 29)		22.5 ± 5.9	23 (19, 27)		550 (27.5)		708 (35.3)	
75-	877 (17.8)	23.2 ± 6.0	24 (20, 28)		20.0 ± 6.8	21 (15, 25)		363 (41.4)		423 (48.2)	
Gender				< 0.0001			< 0.0001		0.1478		0.5845
male	2162 (43.9)	26.0 ± 4.3	27 (24, 29)		23.2 ± 5.5	24 (20, 28)		596 (27.6)		773 (35.8)	
female	2761 (56.1)	25.1 ± 5.2	27 (22, 29)		22.1 ± 6.5	23 (18, 28)		813 (29.5)		1008 (36.5)	
Educational level				< 0.0001			< 0.0001		0.1124		0.0004
below elementary school	1554 (31.6)	23.0 ± 5.9	24 (19, 28)		19.4 ± 7.0	20 (14, 25)		521 (33.5)		545 (35.1)	
elementary school	1211 (24.6)	25.8 ± 4.3	27 (24, 29)		22.6 ± 5.5	23 (19, 27)		228 (18.8)		340 (28.1)	
middle school	1262 (25.6)	26.9 ± 3.4	28 (25, 29)		24.5 ± 4.7	25 (21, 29)		425 (33.7)		572 (45.3)	
high school and above	896 (18.2)	27.5 ± 3.1	29 (26, 30)		25.5 ± 4.0	26 (23, 29)		235 (26.2)		324 (36.2)	
Current employment				< 0.0001			< 0.0001		0.0046		0.0009
yes	849 (17.3)	26.2 ± 4.4	28 (25, 29)		23.6 ± 5.8	25 (20, 28)		209 (24.6)		265 (31.2)	
no	4074 (82.8)	25.4 ± 4.9	27 (23, 29)		22.4 ± 6.1	23 (19, 28)		1200 (29.5)		1516 (37.2)	
Monthly household income per capital (Chinese yuan)				< 0.0001			< 0.0001		< 0.0001		< 0.0001
< 1000	1107 (22.5)	23.1 ± 5.7	24 (20, 28)		19.3 ± 6.3	20 (15, 24)		458 (41.4)		534 (48.2)	
1000–3999	3015 (61.2)	25.9 ± 4.5	27 (24, 29)		23.0 ± 5.8	24 (19, 28)		831 (27.6)		1072 (35.6)	
4000-	801 (16.3)	27.6 ± 3.2	29 (27, 30)		25.8 ± 4.6	27 (23, 30)		120 (15.0)		175 (21.9)	
Residence area				< 0.0001			< 0.0001		< 0.0001		< 0.0001
urban	1321 (26.8)	27.5 ± 3.3	29 (26, 30)		25.7 ± 4.5	27 (23, 30)		261 (19.8)		325 (24.6)	
suburban	1095 (22.2)	26.7 ± 3.4	29 (28, 30)		24.3 ± 4.7	25 (21, 28)		228 (20.8)		312 (28.5)	
county town	1183 (24.0)	25.0 ± 5.3	27 (23, 29)		21.5 ± 6.0	22 (18, 26)		347 (29.3)		482 (40.7)	
village	1324 (26.9)	23.0 ± 5.5	24 (20, 27)		19.1 ± 6.4	19 (15, 24)		573 (43.3)		662 (50.0)	
Current smoking				0.5812			0.3050		0.0261		0.0202
yes	770 (15.6)	25.7 ± 4.2	27 (23, 29)		22.6 ± 5.5	23 (19, 27)		246 (32.0)		307 (39.9)	
no	4153 (84.4)	25.5 ± 5.0	27 (23, 29)		22.6 ± 6.2	23 (19, 28)		1163 (28.0)		1474 (35.5)	
Alcohol intake				< 0.0001			< 0.0001		0.2011		0.6954
yes	843 (17.1)	26.4 ± 3.8	28 (25, 29)		23.6 ± 5.1	24 (20, 28)		226 (26.8)		300 (35.6)	
no	4080 (82.9)	25.3 ± 5.0	27 (23, 29)		22.4 ± 6.3	23 (18, 28)		1183 (29.0)		1481 (36.3)	
Meeting sleep duration recommendation				< 0.0001			< 0.0001		0.0049		0.0031
yes	3346 (68.0)	25.8 ± 4.8	27 (24, 29)		22.9 ± 5.9	24 (19, 28)		916 (27.4)		1164 (34.8)	
no	1577 (32.0)	25.0 ± 4.9	26 (22, 29)		21.9 ± 6.3	23 (18, 27)		493 (31.3)		617 (39.1)	
Hypertension history				< 0.0001			< 0.0001		< 0.0001		< 0.0001
yes	1567 (31.8)	24.8 ± 5.4	26 (22, 29)		21.6 ± 6.5	22 (18, 27)		519 (33.1)		628 (40.1)	
no	3356 (68.2)	25.9 ± 4.5	27 (24, 29)		23.0 ± 5.8	24 (19, 28)		890 (26.5)		1153 (34.4)	
Diabetes history				0.4925			0.5552		0.2211		0.2125
yes	477 (9.7)	25.5 ± 4.7	27 (23, 29)		22.6 ± 5.8	23 (19, 27)		148 (31.0)		185 (38.8)	
no	4446 (90.3)	25.5 ± 4.9	27 (23, 29)		22.6 ± 6.1	23 (19, 28)		1261 (28.4)		1596 (35.9)	
Stroke history				0.6533			0.7690		0.7364		0.5581
yes	96 (2.0)	25.6 ± 4.5	27 (23, 29)		22.9 ± 5.8	24 (19, 28)		26 (27.1)		32 (33.3)	
no	4827 (98.1)	25.5 ± 4.9	27 (23, 29)		22.6 ± 6.1	23 (19, 28)		1383 (28.7)		1749 (36.2)	
Myocardial infarction history				0.6396			0.8370		0.4333		0.4272
yes	93 (1.9)	25.6 ± 4.6	27 (23, 29)		22.9 ± 5.4	24 (20, 26)		30 (32.3)		30 (32.3)	
no	4830 (98.1)	25.5 ± 4.9	27 (23, 29)		22.6 ± 6.1	23 (19, 28)		1379 (28.6)		1751 (36.3)	
Depression				< 0.0001			< 0.0001		0.0013		0.0293
yes	424 (8.6)	24.1 ± 5.7	26 (21, 29)		21.2 ± 7.0	22 (16, 27)		150 (35.4)		174 (41.0)	
no	4499 (91.4)	25.6 ± 4.7	27 (23, 29)		22.7 ± 6.0	23 (19, 28)		1259 (28.0)		1607 (35.7)	
Total^a^	4923 (100)	25.5 ± 4.9	27 (23, 29)		22.6 ± 6.1	23 (19, 28)		1409 (28.6)		1781 (36.2)	

^a^ Percentage of relative standard deviation in total subjects was 19.0 and 26.9% in MMSE and MoCA, respectively, and significant differences (*p* < 0.0001) was found between MMSE and MoCA

### Comparison of cognitive assessment between MMSE and MoCA, and cognitive function by sociodemographic and health-related factors

**Fig. 1:**
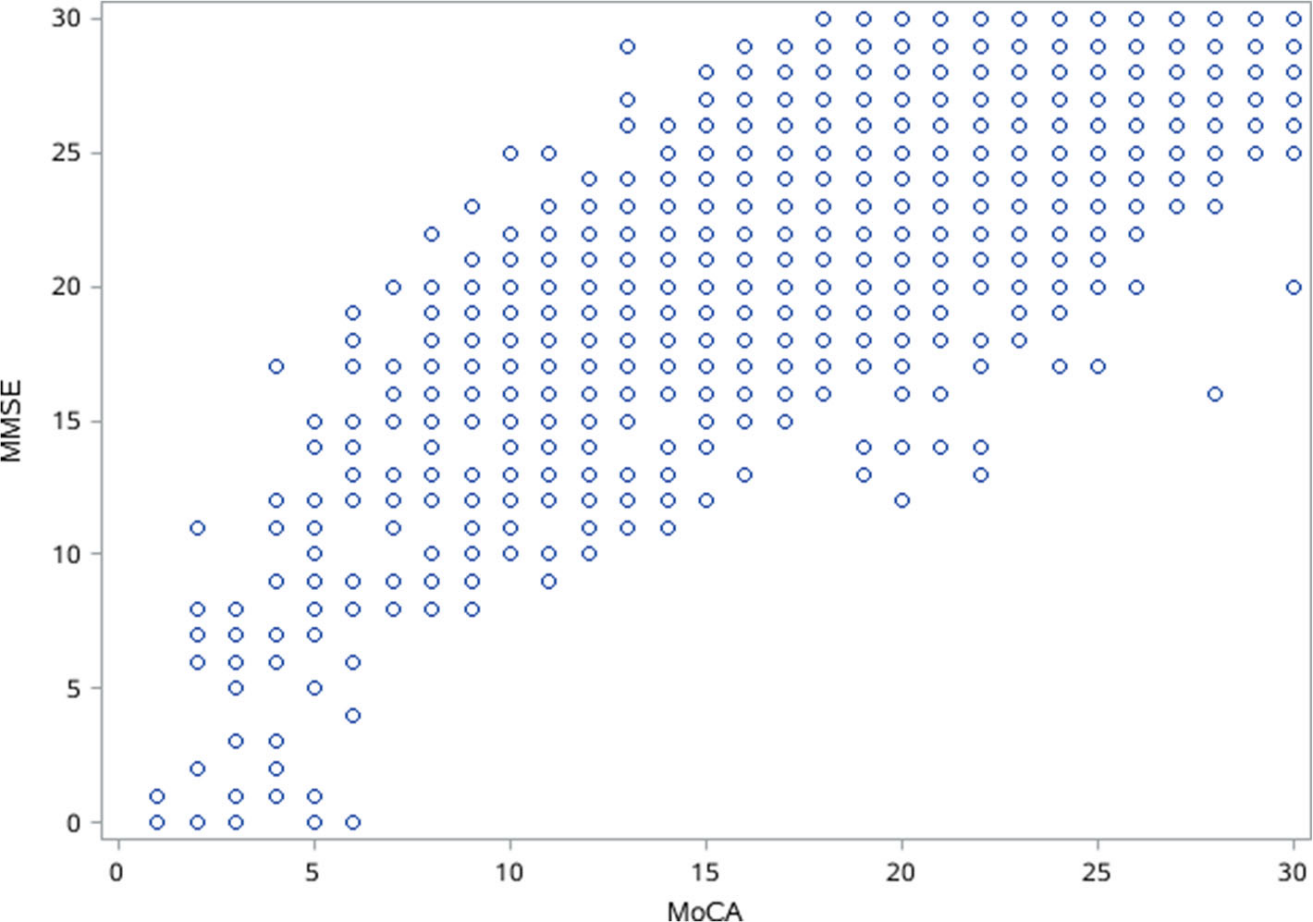
The correlation between the MMSE and MoCA total scores in Chinese population aged ≥55 years

Average score of cognitive test using MMSE and MoCA in total population was 25.5 ± 4.9 and 22.6 ± 6.1, respectively (Table 2), and the MoCA RSD% (26.9%) was significantly greater than that in MMSE (19.0%) (*p* < 0.0001). Scatter plot of Fig. 1 depicted the relationship between MMSE and MoCA total scores, and Spearman correlation coefficient was 0.8374 (*p* < 0.0001).

There were significant differences in the distribution of both MMSE and MoCA total scores by age group, gender, educational level, current employment status, household income, residence area, alcohol intake, sleep duration condition, history of hypertension, and depression (all *p* < 0.0001, Table 2). Multiple comparisons further indicated that total scores of either MMSE or MoCA in subjects aged 55–64 years, those with education of high school and above, and high monthly household income per capital, and those living in urban area, were likely to be the largest among their corresponding subgroups (all *p* < 0.05). Percentile analysis showed presence of ceiling effect (maximum total score on the 75th percentile) for MMSE in several subgroups of 55–64 years, education of high school and above, high level of monthly household income, urban and suburban areas of residence, but for MoCA only in subgroups with high monthly household income level and living in urban area.

According to cutoffs of MCI screening by MMSE and MoCA tests, prevalence of MCI in total population was 28.6 and 36.2%, respectively (Table 2). A total of 1158/4923 (23.5%) subjects fell into MCI for both MMSE and MoCA whereas 623/4923 (12.7%) who tested normal in the MMSE actually tested positive for MCI using MoCA. Of the total sample studied, 2891/4923 (58.7%) had normal scores for both tests (Table 3). The Kappa value indicating agreement for diagnosis of MCI using MoCA versus MMSE was 0.5973 (95% CI: 0.5737, 0.6209) with *p* < 0.0001.

**Table 3:** Agreement of MMSE and MoCA to detect MCI

MMSE	MoCA		
	MCI, n (%)	no MCI, n (%)	Total
MCI, n (%)	1158 (23.5)	251 (5.1)	1409 (28.6)
no MCI, n (%)	623 (12.7)	2891 (58.7)	3514 (71.4)
Total	1781 (36.2)	3142 (63.8)	4923 (100.0)

Significant increased trends in MCI prevalence were observed along with the ascending age groups in both MMSE and MoCA settings (*p* < 0.0001), in which a higher proportion was observed in those ≥75 years (41.4% for MMSE and 48.2% for MoCA), while opposite trends were found in case of household income level (*p* < 0.0001) (Table 2). Prevalence of MCI detected by either MMSE or MoCA was considerably higher in subjects who were unemployed, currently smoked cigarettes, and had inappropriate sleep duration, hypertension history, or depression, compared to their referred groups (all *p* < 0.05). In addition, significant differences in MCI prevalence in setting of MMSE or MoCA were observed among areas of residence. However, only significant differences in MCI prevalence by educational level were found in MoCA test (*p* = 0.0004).

### Subscores of cognitive domains assessed by MMSE and MoCA

**Fig. 2:**
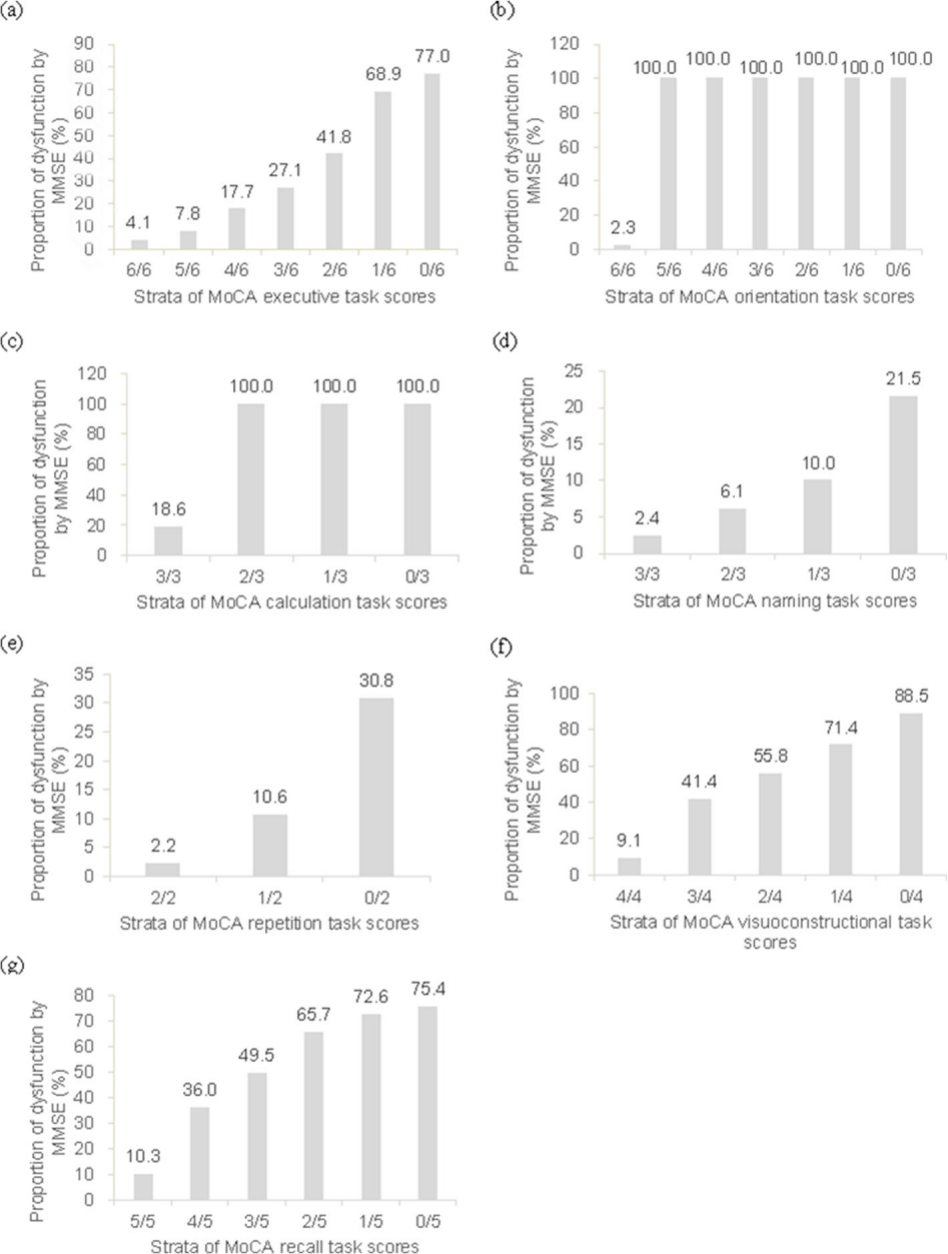
Proportion of subjects with cognitive domain dysfunction by MMSE across strata of MoCA-assessed cognitive subscore. Cognitive domains included **a** executive function, **b** orientation, **c** calculation, **d** naming, **e** repetition, **f** visuoconstructional function, and **g** recall

Based on the distribution of each cognitive domain subscore in total samples assessed by different items of MMSE and MoCA (Table 4), the performance of execution, repetition and registration among 75% subjects using MMSE met maximum scores, whereas executive and recall dysfunctions were found in about 75% participants by MoCA test. The function of naming was performed well in both scales. Present study further focused on cognitive domains tested by both MMSE and MoCA, and found significant increased trends in proportions of subjects with cognitive dysfunction in terms of orientation, execution, calculation, naming, repetition, visuoconstruction and recall by MMSE across strata of the corresponding cognitive domain score by MoCA (all *p* < 0.0001, Fig. 2).

**Table 4:** Subscores of different cognitive domains by MMSE and MoCA in total subjects

Domains	MMSE			MoCA		
	Items/maximum scores	mean ± SD	P_50_ (P_25_, P_75_)	Items/maximum scores	mean ± SD	P_50_ (P_25_, P_75_)
Orientation	Orientation to time and place/10	9.4 ± 1.4	10 (9, 10)	Orientation to time and place/6	5.5 ± 1.1	6 (5, 6)
Executive function	3-step command test/3	3.6 ± 0.9	4 (4, 4)	Trail-making test/1	3.7 ± 1.7	4 (2, 5)
	Reading command test/1			Digit span test/2		
				Verbal fluency/1		
				Abstraction test/2		
Calculation	Serial 7 substractions/5	3.7 ± 1.7	5 (2, 5)	Serial 7 substractions/3	2.4 ± 0.9	3 (2, 3)
Naming	Naming (pencil, watch)/2	1.9 ± 0.3	2 (2, 2)	Naming (lion, giraffe, camel)/3	2.7 ± 0.7	3 (3, 3)
Repetition	1 short sentence/1	0.9 ± 0.3	1 (1, 1)	2 longer sentences/2	1.2 ± 0.8	1 (0, 2)
Visuoconstructional skills	Copy intersecting pentagons/1	0.6 ± 0.5	1 (0, 1)	Copy cube/1	2.6 ± 1.4	3 (2, 4)
				Draw clock face/3		
Registration	Repeat 3 words/3	2.7 ± 0.7	3 (3, 3)		na	na
Recall	Recall 3 words/3	2.3 ± 1.0	3 (2, 3)	Recall 5 words/5	3.0 ± 1.7	3 (2, 5)
Writing	Write a sentence/1	0.5 ± 0.5	1 (0, 1)		na	na
Attention		na	na	Vigilance test for number ‘1’/1	0.6 ± 0.5	1 (0, 1)

*na* not available

### Potential factors associated with MCI risk detected by MMSE and MoCA

Sociodemographic and health-related factors associated with MCI risk by multiple logistic regression model were highly consistent between MMSE and MoCA scales (Table 5). Especially, subjects aged ≥75 years (OR = 2.073, 95% CI: 1.727, 2.489 for MMSE; OR = 1.869, 95% CI: 1.570, 2.227 for MoCA) significantly increased the risk of MCI compared to the reference of 55–64 years. The odds of MCI in females was 28.0% in MMSE and 16.3% in MoCA greater than that in males. Being employed currently and living in a family with moderate or high monthly household income per capital highly reduced risk of MCI in both scales relative to their respective control group (all *p* < 0.05). Current smoking was identified as a risk factor with a 37.3 and 28.8% higher odds of MCI by MMSE and MoCA, respectively, compared to no smoking. A higher likelihood of MCI was observed in subjects living in county town or village, and those with hypertension history or self-reported depression (all *p* < 0.05).

**Table 5:** Potential factors associated with the risk of MCI using multiple logistic regression model^a^

Predictors	MCI by MMSE			MCI by MoCA		
	Coefficient β	Adjusted OR (95% CI)	*p*-value	Coefficient β	Adjusted OR (95% CI)	*p*-value
Age group (years)						
65–74	0.0916	1.096 (0.944, 1.273)	0.2295	0.0840	1.088 (0.947, 1.249)	0.2343
75-	0.7291	2.073 (1.727, 2.489)	< 0.0001	0.6256	1.869 (1.570, 2.227)	< 0.0001
Gender (females)	0.2472	1.280 (1.106, 1.485)	0.0010	0.1508	1.163 (1.014, 1.334)	0.0308
Current employment (yes)	−0.3203	0.726 (0.600, 0.876)	0.0009	−0.3386	0.713 (0.597, 0.849)	0.0002
Monthly household income per capital (Chinese yuan)						
1000–3999	− 0.3387	0.713 (0.608, 0.835)	< 0.0001	− 0.2035	0.816 (0.700, 0.950)	0.0089
4000-	−0.8954	0.408 (0.316, 0.526)	< 0.0001	−0.6360	0.529 (0.420, 0.666)	< 0.0001
Residence area						
suburban	−0.0109	0.989 (0.805, 1.214)	0.9172	0.1602	1.174 (0.974, 1.414)	0.0917
county town	0.3262	1.386 (1.132, 1.697)	0.0016	0.6217	1.862 (1.546, 2.244)	< 0.0001
village	0.9476	2.579 (2.125, 3.136)	< 0.0001	1.0012	2.721 (2.266, 3.273)	< 0.0001
Current smoking (yes)	0.3169	1.373 (1.128, 1.669)	0.0015	0.2535	1.288 (1.072, 1.548)	0.0068
Meeting sleep duration recommendation (yes)	0.0031	1.003 (0.871, 1.156)	0.9658	−0.0480	0.953 (0.835, 1.089)	0.4785
Hypertension history (yes)	0.2452	1.278 (1.113, 1.466)	0.0005	0.1893	1.208 (1.061, 1.376)	0.0044
Depression (yes)	0.3821	1.465 (1.167, 1.836)	0.0009	0.3000	1.350 (1.085, 1.676)	0.0068

^a^Multiple logistic regression model estimated the risk of MCI associated with potentially independent factors. The reference groups for comparison were 55 ≤ age ≤ 64 years, males, no job currently, monthly per capital income < 1000 Chinese yuan, urban area of residence, no smoking currently, not meeting sleep duration recommendation, no history of hypertension, and no depression, respectively

## Discussion

MCI is a common condition in the elderly, characterized by deterioration of memory, attention, and cognitive function that is beyond what is expected based on age and educational level, but without significant interference with ability of daily activity [35]. Present study found that MCI prevalence in Chinese population aged ≥55 years from urban and rural areas of four provinces using MMSE and MoCA was 28.6 and 36.2%, respectively, and MMSE had good correlation with MoCA (Spearman correlation coefficient = 0.8374) and moderate agreement for detecting MCI with Kappa value of 0.5973. Moreover, increasing age, female, living in county town/village, smoking, hypertension and depression significantly increased the risk of MCI in both tests. All findings indicated serious condition of cognitive impairment along with progressive increase in the growth rate of aging population in China and huge challenges on the prevention and treatment of MCI to the society and government.

The MMSE is the most widely used cognitive screening test by physicians and researchers for general cognitive evaluation [2]. One problem with the MMSE is its ceiling effect or limited dynamic performance range for normal individuals, which increases the likelihood that persons in predementia stages score within the normal range [36]. Consistent with previous study [36], the ceiling effect (28–30 points) for MCI was less using MoCA (26.2%) versus MMSE (46.3%) in this study as clearly depicted in Fig. 1 as well as the distributions of both test scores in Table 2. The greater RSD% in MoCA (26.9%) relative to that in MMSE (19.0%) further suggested MoCA distributed samples across a broader score range with less ceiling effect and had better detection of cognitive heterogeneity of the sample [33]. On the other hand, MoCA was developed by Nasreddine in 2005 as a brief tool to screen subjects who present with cognitive complaints and usually have normal MMSE scores [8]. Here, 12.7% of total subjects with a normal MMSE score actually tested positive for MCI according to MoCA’s adjusted cutoff points, partly reflecting higher sensitivity for MCI in MoCA although no comparison with the gold standard method was performed. This study further focused on cognitive domain subtests by MMSE and MoCA. The observed significantly increased likelihood of incorrect MMSE executive, naming, repetition, visuoconstructional, and recall tasks across decreasing scores of MoCA corresponding tasks (Fig. 2), suggested the higher sensitivity of the MoCA in detecting dysfunctions of abovementioned cognitive domains, which may be related to more components of each domain in MoCA [28]. Together, as indicated above, the MoCA is a better measure to screen for cognitive impairment in middle-aged and older Chinese living in communities relative to MMSE as it lacks ceiling effect and shows better sensitivity.

This study found a high strength correlation between MoCA and MMSE scores with a Spearman correlation coefficient of 0.8374. This positive relationship was highly close to that reported by the original MoCA norms study in older Chinese [27], which obtained good correlation between both tools with Spearman correlation coefficient of 0.83. Both consistently demonstrated adequate level of concurrent validity between MoCA Beijing version and the Chinese version of MMSE for community dwellers. Significant positive correlation between total scores of MoCA and MMSE was also found in the assessment of cognitive deficit associated with chronic diseases [37, 38]. The MoCA and MMSE had a Kappa value of 0.5973, indicating moderate agreement [39]. And the agreement disparity could attribute to the difference in the functions of the instruments themselves, in which MoCA was developed in particular for MCI screening [8] whereas MMSE was originally invented as a tool to detect and monitor the development of dementia [7, 39].

Changes in criteria and differences in populations studied and methodology have produced a wide range of prevalence estimates for MCI. Previous study applied uniform diagnostic criteria to harmonize data from USA, Europe, Asia and Australia, in which MCI prevalence ranged from 5 to 36.7%, and more reliably estimate MCI prevalence, as a result, a reduced MCI prevalence (2.1–20.7%) was produced when using MMSE score of 24–27 to define MCI [40]. Present study found that prevalence of MCI in Chinese aged 55 years and older was 28.6 and 36.2% overall using education-specific cutoffs of MMSE and MoCA, respectively. Studies in mainland China over the past 5 years that used different diagnostic criteria showed MCI prevalence ranging from 12.6 to 34.1% in old population [22, 41–44], and all these studies were conducted in single region, conversely our study covered urban and rural areas in four provinces. Representatively, Jia et al. (2014) reported that prevalence of MCI was 20.8% for individuals aged 65 years and above across multiple regions in China [45]. His group (2020) recently conducted a large national study across different socioeconomic and geographic regions in 12 provinces and municipalities in China and found that the overall MCI prevalence was estimated at 15.5% in people aged 60 years or older, representing 38.77 million people nationwide [46]. We also paid attention to the prevalence of MCI by demographic and health-related factors. Similar to the large-scale study [46], the prevalence of MCI increased with older age, and the higher prevalence of MCI was correlated with rural residence, smoking and hypertension in both MMSE and MoCA instruments in present study (Table 2). Educational level was believed to be the strongest noncognitive factor affecting cognitive test score [27]. Consistently, less education profoundly correlated with poorer performance of the MMSE and MoCA, showing a significant increased trend of MMSE/MoCA score with high education in this study. The results supported the findings of better performance on MoCA for those with 6 years and more education compared to those with less than 6 years education [23]. Oppositely, in the study by Ng et al. [24], education influenced MoCA’s test performance in unpredictable manner, those with more education performed poorer relative to those with less education, which was likely to attribute to tests of MoCA domains of naming, attention, language, abstraction, and orientation. Unexpectedly, people with higher educational level (middle school/high school and above) had a greater prevalence of MCI detected by MoCA. Educational level is one of indicators of cognitive reserve, which influences the manifestation of symptoms of cognitive impairment [47]. People with low education theoretically display a steeper cognitive decline early in the process of aging compared to those with high level of education. Our conflicting findings might indicate that confounders such as the passion for cognitive activity and strong district-level social network may buffer the relationship between low education and cognitive impairment [48]. This raises the need for further study to test MCI by education. We also found other factors associated with MCI, such as household income, employment status, sleep duration and depression. Overall, the high level of variability in reported MCI prevalence worldwide or nationwide may be associated with ethnic and/or regional differences, and the heterogeneity in research methods, including the use of different diagnostic criteria, and the focus of samples with different characteristics, such as age brackets, gender and educational attainment [1]. Anyhow, these findings suggested that MCI is becoming increasingly prevalent all over the world along with the changes in lifestyle and lifespan of human beings, and the clarification of risk factors for MCI would inform specific control measures as many risk factors are modifiable.

MCI is thought to be a transitional stage between being cognitively unimpaired and dementia, consensus has been reached to focus primary intervention on this population to halt dementia progression. With the increasing attention being paid to MCI, studies have been conducted in recent years in a variety of research settings to understand its influencing factors. We conducted systematic assessment of risk factors for MCI, to some extent, including demographic factors, lifestyle, psychological factors and cardiovascular risk factors. Compare to each reference group, increasing age (≥75 years), female gender, living in less urbanized areas (county town or village), current smoking, hypertension and depression considerably increased the odds of MCI detected by both MMSE and MoCA after adjustment for covariates, as reported in previous studies [19, 22, 46, 49, 50]. Among these factors, there is no consensus on the question whether depression is the consequence or the cause for cognitive impairment in older people, but the association between depression and MCI may result in a faster progression of cognitive decline [51]. In contrast, current employment and higher monthly household income per capital (1000–3999 and ≥ 4000 Chinese yuan) were significantly associated with lower risk of MCI, relative to unemployment and less than 1000 Chinese yuan of monthly income in present study, respectively, which was similar to previous findings also conducted in Chinese population [19, 22]. And the protective role of employment was attributed to increased reserve and the ability to tolerate higher levels of neuropathology thereby maintained their cognitive functioning [2], on the other hand, employment status would get access to higher social engagement, which was beneficial for MCI prevention [2, 19].

There were several limitations in this study. First, the Chinese version of MMSE and MoCA scales and accordingly education-specific cutoffs of MCI were used in this study, which partly affected international comparison of prevalence rate and influencing factors of MCI. Second, due to limited data, we cannot analyze the impact of dietary intakes and genetic factors on cognitive impairment in this population. Additionally, a gold standard was not employed to detect MCI, as a result, this study failed to compare the sensitivity and specificity between MMSE and MoCA. Finally, false positive and false negative existed in MCI screening.

## Conclusions

The findings of this study showed that MMSE and MoCA had good correlation and moderate agreement for detecting MCI in Chinese population aged 55 years and above. But MoCA had less ceiling effect for MCI and better detection of cognitive heterogeneity of the sample. High overall MCI prevalence was observed in both screenings, and residence of county town and village, current smoking, hypertension and depression were identified as modifiable risk factors for MCI except for increasing age, female gender. The cognitive function of the elderly will experience inevitable deterioration in China with the rapidly increasing aging population in near future, which poses a huge challenge for public health system and medical nursing system in China. Taken together, these findings indicate severe status of MCI in Chinese old population and provide important evidence for the establishment of specific intervention measures. Increasing public awareness of MCI and dementia, controlling MCI risk factors to delay dementia onset and boosting the implement of established strategies by authorities would effectively reduce the prevalence of MCI and dementia in China.

## Data Availability

The datasets used and analysed during the current study are available from the corresponding author on reasonable request.

## Abbreviations

AD: Alzheimer’s disease
GDS: Geriatric depression scale
MCI: Mild cognitive impairment
MMSE: Mini-Mental State Examination
MoCA: Montreal Cognitive Assessment
RSD: Relative standard deviation
SD: Standard deviation

## References

[1] Jia L, Quan M, Fu Y, Zhao T, Li Y, Wei C, Tang Y, Qin Q, Wang F, Qiao Y, Shi S, Wang YJ, du Y, Zhang J, Zhang J, Luo B, Qu Q, Zhou C, Gauthier S, Jia J, Group for the Project of Dementia Situation in China (2020). Dementia in China: epidemiology, clinical management, and research advances. Lancet Neurol.

[2] Anderson ND (2019). State of the science on mild cognitive impairment (MCI). CNS Spectr.

[3] Morley JE (2018). An overview of cognitive impairment. Clin Geriatr Med.

[4] Sanford AM (2017). Mild cognitive impairment. Clin Geriatr Med.

[5] Hawkins MA, Gathright EC, Gunstad J, Dolansky MA, Redle JD, Josephson R (2014). The MoCA and MMSE as screeners for cognitive impairment in a heart failure population: a study with comprehensive neuropsychological testing. Heart Lung.

[6] Lim MYL, Loo JHY (2018). Screening an elderly hearing impaired population for mild cognitive impairment using Mini-mental state examination (MMSE) and Montreal cognitive assessment (MoCA). Int J Geriatr Psychiatry..

[7] Folstein MF, Folstein SE, McHugh PR (1975). “Mini-mental state”. A practical method for grading the cognitive state of patients for the clinician. J Psychiatr Res.

[8] Nasreddine ZS, Phillips NA, Bédirian V, Charbonneau S, Whitehead V, Collin I (2005). The Montreal cognitive assessment, MoCA: a brief screening tool for mild cognitive impairment. J Am Geriatr Soc.

[9] Ciesielska N, Sokolowski R, Mazur E, Podhorecka M, Polak-Szabela A, Kedziora-Kornatowska K (2016). Is the Montreal cognitive assessment (MoCA) test better suited than the Mini-mental state examination (MMSE) in mild cognitive impairment (MCI) detection among people aged over 60? Meta-analysis. Psychiatr Pol.

[10] Dong Y, Lee WY, Basri NA, Collinson SL, Merchant RA, Venketasubramanian N, Chen CLH (2012). The Montreal cognitive assessment is superior to the Mini-mental state examination in detecting patients at higher risk of dementia. Int Psychogeriatr.

[11] Larner AJ (2012). Screening utility of the Montreal cognitive assessment (MoCA): in place of--or as well as--the MMSE?. Int Psychogeriatr.

[12] Breton A, Casey D, Arnaoutoglou NA (2019). Cognitive tests for the detection of mild cognitive impairment (MCI), the prodromal stage of dementia: Meta-analysis of diagnostic accuracy studies. Int J Geriatr Psychiatry.

[13] Pinto TCC, Machado L, Bulgacov TM, Rodrigues-Junior AL, Costa MLG, Ximenes RCC (2019). Is the Montreal cognitive assessment (MoCA) screening superior to the Mini-mental state examination (MMSE) in the detection of mild cognitive impairment (MCI) and Alzheimer’s disease (AD) in the elderly?. Int Psychogeriatr.

[14] Pendlebury ST, Markwick A, de Jager CA, Zamboni G, Wilcock GK, Rothwell PM (2012). Differences in cognitive profile between TIA, stroke and elderly memory research subjects: a comparison of the MMSE and MoCA. Cerebrovasc Dis.

[15] Siqueira GSA, Hagemann PMS, Coelho DS, Santos FHD, Bertolucci PHF (2019). Can MoCA and MMSE be interchangeable cognitive screening tools? A Systematic Review. Gerontologist.

[16] Dong Y, Yean Lee W, Hilal S, Saini M, Wong TY, Chen CL (2013). Comparison of the Montreal cognitive assessment and the Mini-mental state examination in detecting multi-domain mild cognitive impairment in a Chinese sub-sample drawn from a population-based study. Int Psychogeriatr.

[17] Cao L, Hai S, Lin X, Shu D, Wang S, Yue J, Liu G, Dong B (2012). Comparison of the Saint Louis University mental status examination, the Mini-mental state examination, and the Montreal cognitive assessment in detection of cognitive impairment in Chinese elderly from the geriatric department. J Am Med Dir Assoc.

[18] Zhou DF, Wu CS, Qi H, Fan JH, Sun XD, Como P, Qiao YL, Zhang L, Kieburtz K (2006). Prevalence of dementia in rural China: impact of age, gender and education. Acta Neurol Scand.

[19] Zhang Q, Wu Y, Han T, Liu E (2019). Changes in cognitive function and risk factors for cognitive impairment of the elderly in China: 2005-2014. Int J Environ Res Public Health.

[20] Xiu S, Zheng Z, Liao Q, Chan P (2019). Different risk factors for cognitive impairment among community-dwelling elderly, with impaired fasting glucose or diabetes. Diabetes Metab Syndr Obes.

[21] Xiu S, Liao Q, Sun L, Chan P (2019). Risk factors for cognitive impairment in older people with diabetes: a community-based study. Ther Adv Endocrinol Metab.

[22] Ren L, Zheng Y, Wu L, Gu Y, He Y, Jiang B, Zhang J, Zhang L, Li J (2018). Investigation of the prevalence of cognitive impairment and its risk factors within the elderly population in Shanghai, China. Sci Rep.

[23] Din NC, Shahar S, Zulkifli BH, Razali R, Vyrn CA, Omar A (2016). Validation and optimal cut-off scores of the Bahasa Malaysia version of the Montreal cognitive assessment (MoCA-BM) for mild cognitive impairment among community dewelling older adults in Malaysia. Sains Malaysiana.

[24] Ng TP, Feng L, Lim WS, Chong MS, Lee TS, Yap KB, Tsoi T, Liew TM, Gao Q, Collinson S, Kandiah N, Yap P (2015). Montreal cognitive assessment for screening mild cognitive impairment: variations in test performance and scores by education in Singapore. Dement Geriatr Cogn Disord.

[25] Huang Q, Jia X, Zhang J, Huang F, Wang H, Zhang B, Wang L, Jiang H, Wang Z (2021). Diet-cognition associations differ in mild cognitive impairment subtypes. Nutrients..

[26] Katzman R, Zhang MY, Ouang Ya Q, Wang ZY, Liu WT, Yu E (1988). A Chinese version of the Mini-mental state examination; impact of illiteracy in a Shanghai dementia survey. J Clin Epidemiol.

[27] Lu J, Li D, Li F, Zhou A, Wang F, Zuo X, Jia XF, Song H, Jia J (2011). Montreal cognitive assessment in detecting cognitive impairment in Chinese elderly individuals: a population-based study. J Geriatr Psychiatry Neurol.

[28] Fu C, Jin X, Chen B, Xue F, Niu H, Guo R, Chen Z, Zheng H, Wang L, Zhang Y (2017). Comparison of the Mini-mental state examination and Montreal cognitive assessment executive subtests in detecting post-stroke cognitive impairment. Geriatr Gerontol Int.

[29] Zhang Z, Hong X, Li H, Zhao J, Huang J, Jing W (1999). The mini-mentai state examination in Chinese residentrs population aged 55 years and over in the urban and rural areas of Beijing. Chin J Neurol.

[30] Yesavage JA, Brink TL, Rose TL, Lum O, Huang V, Adey M, Leirer VO (1982). Development and validation of a geriatric depression screening scale: a preliminary report. J Psychiatr Res.

[31] Brink TL, Yesavage JA, Lum O, Heersema PH, Adey M, Rose TL (1982). Screening tests for geriatric depression. Clin Gerontol.

[32] Hirshkowitz M, Whiton K, Albert SM, Alessi C, Bruni O, DonCarlos L, Hazen N, Herman J, Katz ES, Kheirandish-Gozal L, Neubauer DN, O’Donnell AE, Ohayon M, Peever J, Rawding R, Sachdeva RC, Setters B, Vitiello MV, Ware JC, Adams Hillard PJ (2015). National Sleep Foundation’s sleep time duration recommendations: methodology and results summary. Sleep Health.

[33] Biundo R, Weis L, Bostantjopoulou S, Stefanova E, Falup-Pecurariu C, Kramberger MG, Geurtsen GJ, Antonini A, Weintraub D, Aarsland D (2016). MMSE and MoCA in Parkinson’s disease and dementia with Lewy bodies: a multicenter 1-year follow-up study. J Neural Transm (Vienna).

[34] Miyawaki CE, Liu M (2019). Gender differences in cognitive impairment among the old and the oldest-old in China. Geriatr Gerontol Int.

[35] Eshkoor SA, Hamid TA, Mun CY, Ng CK (2015). Mild cognitive impairment and its management in older people. Clin Interv Aging.

[36] Trzepacz PT, Hochstetler H, Wang S, Walker B, Saykin AJ, Alzheimer’s disease neuroimaging I (2015). Relationship between the Montreal Cognitive Assessment and Mini-mental State Examination for assessment of mild cognitive impairment in older adults. BMC Geriatr.

[37] Fisekovic S, Memic A, Pasalic A (2012). Correlation between Moca and mmse for the assessment of cognition in schizophrenia. Acta Inform Med.

[38] Tiffin-Richards FE, Costa AS, Holschbach B, Frank RD, Vassiliadou A, Kruger T (2014). The Montreal cognitive assessment (MoCA) - a sensitive screening instrument for detecting cognitive impairment in chronic hemodialysis patients. PLoS One.

[39] Razali R, Jean-Li L, Jaffar A, Ahmad M, Shah SA, Ibrahim N, Din NC, Jaafar NRN, Midin M, Sidi H, Ahmad S (2014). Is the Bahasa Malaysia version of the Montreal cognitive assessment (MoCA-BM) a better instrument than the Malay version of the Mini mental state examination (M-MMSE) in screening for mild cognitive impairment (MCI) in the elderly?. Compr Psychiatry.

[40] Arendt T, Sachdev PS, Lipnicki DM, Kochan NA, Crawford JD, Thalamuthu A (2015). The prevalence of mild cognitive impairment in diverse geographical and Ethnocultural regions: the COSMIC collaboration. PLoS One.

[41] Giri M, Chen T, Yu W, Lü Y (2016). Prevalence and correlates of cognitive impairment and depression among elderly people in the world’s fastest growing city, Chongqing, People’s republic of China. Clin Interv Aging.

[42] Liu X, Yin X, Tan A, He M, Jiang D, Hou Y, Lu Y, Mao Z (2018). Correlates of mild cognitive impairment of community-dwelling older adults in Wuhan, China. Int J Environ Res Public Health.

[43] Lu H, Wang X-D, Shi Z, Yue W, Zhang Y, Liu S, Liu S, Zhao L, Xiang L, Zhang Y, Guan Y, Su W, Li Z, Wang J, Wisniewski T, Ji Y (2019). Comparative analysis of cognitive impairment prevalence and its etiological subtypes in a rural area of northern China between 2010 and 2015. Sci Rep.

[44] Rao D, Luo X, Tang M, Shen Y, Huang R, Yu J, Ren J, Cheng X, Lin K (2018). Prevalence of mild cognitive impairment and its subtypes in community-dwelling residents aged 65 years or older in Guangzhou, China. Arch Gerontol Geriatr.

[45] Jia J, Zhou A, Wei C, Jia X, Wang F, Li F, Wu X, Mok V, Gauthier S, Tang M, Chu L, Zhou Y, Zhou C, Cui Y, Wang Q, Wang W, Yin P, Hu N, Zuo X, Song H, Qin W, Wu L, Li D, Jia L, Song J, Han Y, Xing Y, Yang P, Li Y, Qiao Y, Tang Y, Lv J, Dong X (2014). The prevalence of mild cognitive impairment and its etiological subtypes in elderly Chinese. Alzheimers Dement.

[46] Jia L, Du Y, Chu L, Zhang Z, Li F, Lyu D (2020). Prevalence, risk factors, and management of dementia and mild cognitive impairment in adults aged 60 years or older in China: a cross-sectional study. Lancet Public Health.

[47] Lojo-Seoane C, Facal D, Juncos-Rabadán O (2012). Does intellectual activity prevent cognitive impairment? Relationships between cognitive reserve and mild cognitive impairment. Rev Esp Geriatr Gerontol.

[48] Murayama H, Miyamae F, Ura C, Sakuma N, Sugiyama M, Inagaki H, Okamura T, Awata S (2019). Does community social capital buffer the relationship between educational disadvantage and cognitive impairment? A multilevel analysis in Japan. BMC Public Health.

[49] Lu Y, An Y, Guo J, Zhang X, Wang H, Rong H, Xiao R (2016). Dietary intake of nutrients and lifestyle affect the risk of mild cognitive impairment in the Chinese elderly population: a cross-sectional study. Front Behav Neurosci.

[50] Lee CH, Kim DH, Moon YS (2019). Differential associations between depression and cognitive function in MCI and AD: a cross-sectional study. Int Psychogeriatr.

[51] Defrancesco M, Marksteiner J, Deisenhammer EA, Hinterhuber H, Weiss EM (2009). Association of Mild Cognitive Impairment (MCI) and depression. Neuropsychiatr..

